# Different endurance exercises modulate NK cell cytotoxic and inhibiting receptors

**DOI:** 10.1007/s00421-021-04735-z

**Published:** 2021-09-03

**Authors:** A. Pal, J. Schneider, K. Schlüter, K. Steindorf, J. Wiskemann, F. Rosenberger, P. Zimmer

**Affiliations:** 1 Division of Physical Activity, Prevention and Cancer, German Cancer Research Center (DKFZ) and National Center for Tumor Diseases (NCT), Heidelberg, Germany; 2 Medical Faculty Heidelberg, University of Heidelberg, Im Neuenheimer Feld 581, 69120 Heidelberg, Germany; 3 Department of Medical Oncology, National Center for Tumor Diseases (NCT), Heidelberg University Hospital, Im Neuenheimer Feld 460, 69120 Heidelberg, Germany; 4 Institute of Sports and Sport Science, Heidelberg University, Seminarstrasse 1, 69117 Heidelberg, Germany; 5 Institute for Sport and Sport Science, Division of “Performance and Health (Sports Medicine)” , TU Dortmund University, August-Schmidt-Straße 4, 44227 Dortmund, Germany

**Keywords:** Exercise, Physical activity, Cancer, IDO/TDO, Kynurenine, Tryptophan, NK Cell

## Abstract

**Purpose:**

Induction of IDO depends on the activation of AhR forming the AhR/IDO axis. Activated AhR can transcribe various target genes including cytotoxic and inhibiting receptors of NK cells. We investigated whether AhR and IDO levels as well as activating (NKG2D) and inhibiting (KIR2DL1) NK cell receptors are influenced by acute exercise and different chronic endurance exercise programs.

**Methods:**

21 adult breast and prostate cancer patients of the TOP study (NCT02883699) were randomized to intervention programs of 12 weeks of (1) endurance standard training or (2) endurance polarized training after a cardiopulmonary exercise test (CPET). Serum was collected pre-CPET, immediately post-CPET, 1 h post-CPET and after 12 weeks post-intervention. Flow cytometry analysis was performed on autologous serum incubated NK-92 cells for: AhR, IDO, KIR2DL1 and NKG2D. Differences were investigated using analysis-of-variance for acute and analysis-of-covariance for chronic effects.

**Results:**

Acute exercise: IDO levels changed over time with a significant increase from post-CPET to 1 h post-CPET (*p* = 0.03). KIR2DL1 levels significantly decreased over time (*p* < 0.01). NKG2D levels remained constant (*p* = 0.31). Chronic exercise: for both IDO and NKG2D a significant group × time interaction, a significant time effect and a significant difference after 12 weeks of intervention were observed (IDO: all *p* < 0.01, NKG2D: all *p* > 0.05).

**Conclusion:**

Both acute and chronic endurance training may regulate NK cell function via the AhR/IDO axis. This is clinically relevant, as exercise emerges to be a key player in immune regulation.

**Supplementary Information:**

The online version contains supplementary material available at 10.1007/s00421-021-04735-z.

## Introduction

A growing body of evidence suggests positive effects of physical exercise on cancer development, progress and mortality (Campbell et al. 2019; Patel et al. 2019; Meyerhardt et al. 2006). One mechanism which was hypothesized to promote these beneficial effects is an exercise-induced mobilisation and activation of tumor-competitive lymphocytes, such as natural killer cells (NK cells) (Idorn and Hojman 2016).

As part of the innate immune system, NK cells can recognize and eliminate virus-infected and neoplastic cells. NK cells cytotoxic ability is complex and is regulated by a range of activating and inhibitory receptors expressed on the NK cell surface (Jones et al. 2012; Kumar 2018). A balance of these activating and inhibitory signals mediated by these receptors determines whether NK cell responses will proceed (Campbell and Purdy 2011). One of the dominant activating receptors on NK cells is NKG2D. In the context of cancer cell surveillance and removal, NKG2D binds to ligands of cellular stress often overexpressed on malignantly transformed cells triggering cytokine secretion or direct cellular cytotoxicity (Schmiedel and Mandelboim 2018). Within the inhibitory cascade, the killer cell immunoglobulin-like receptor (KIR) family is a dominant group of negative regulators which binds to self MHC- class I ligands (HLA-A, -B, -C) (Kumar 2018).

For immune surveillance and elimination of transformed cells it is important for NK cells to be mobilized and cytotoxic. There is a direct relationship between NK cell deployment and catecholamine release during and immediately after exercise. Studies have revealed that acute exercise leads to NK cell mobilization by release of epinephrine binding to their β andrenergic receptors (Pedersen et al. 2016). A recent meta-analysis reported a significant and strong increase in NK cell cytotoxic activity after acute exercise (Rumpf et al. 2020). In contrast to the acute effects mentioned above, there exists limited and contradictory literature regarding the effects of chronic exercise training on NK cell mobilization or function (Mohamady et al. 2013; Fairey et al. 2005; Nieman et al. 1990, 1993). Apart from that, knowledge about the underlying mechanisms provoking acute and chronic changes in NK cell cytotoxicity is still sparse.

**Fig. 1:**
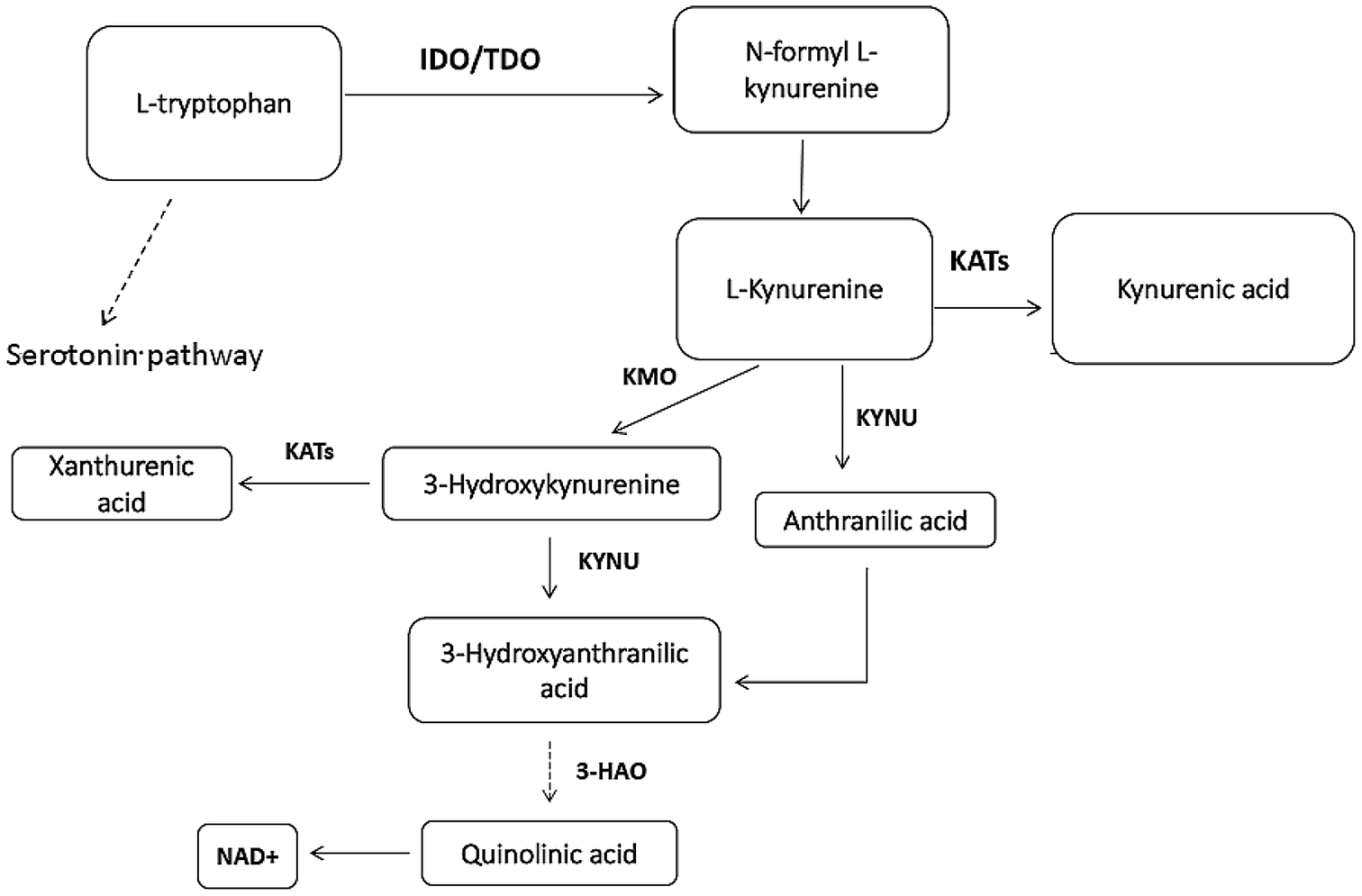
Major metabolites of the Tryptophan metabolism pathway in humans. *IDO* indoleamine 2,3-dioxygenase; *TDO* Tryptophan 2,3-dioxygenase; *KAT* KYN aminotrasferase I; *KMO* KYN monooxygenase; *3-HK* 3-hudroxyKynurenine; *KYNU* Kynureninase (Pal et al. 2021)

The Kynurenine pathway has emerged to be one such mechanism that has an extensive role in immuno-modulation (Heng et al. 2016). Stimulated by inflammation, the enzyme IDO (indolamine 2,3-dioxegenase 1 or 2) converts Tryptophan to Kynurenine. IDO-1 is the predominant isoform catalysing the rate-limiting step of Kynurenine formation (Badawy 2017). Several investigations have revealed that the proliferation and function of NK cells can be suppressed by IDO and its products (Fig. 1) (Kai et al. 2003).

Recent work showed that IDO induction depends on activation of the Aryl hydrocarbon receptor (AhR) (Nguyen et al. 2014). AhR is a ligand-activated member of transcription factors with Kynurenine and Kynurenic Acid as a potent internal AhR agonists (Kerkvliet 2012; Sogawa and Fujii-Kuriyama 1997). When AhR binds to its ligands, the AHR-ligand complex translocates to the nucleus and binds AHR nuclear translocator (Arnt) (Burbach et al. 1992; Schrenk 1998). The AhR–Arnt heterodimers bind specific motifs, called dioxin-responsive elements (DREs), in the promoter region of target genes. These target genes include a wide array of cytotoxic and inhibiting receptor codes, e.g., NKG2D, KIRs etc. (Park et al. 2019).

The impact of exercise on the initial step of the Kynurenine pathway, namely IDO/TDO (Tryptophan 2,3-dioxygenase) expression and activity is poorly investigated. In healthy populations, it has been shown that after an acute bout of exercise, plasma Tryptophan levels decrease, whereas plasma Kynurenine levels increase (Strasser et al. 2016a, b). In disease populations, few studies have investigated the effects of acute or chronic exercise from low to moderate intensities on Tryptophan and Kynurenine levels (Bansi et al. 2018; Hennings et al. 2013; Millischer et al. 2017). We have recently shown that exercise can counteract radiation-associated increase in Kynurenine levels and IDO/TDO activity in breast cancer patients as well as reduce IDO (serum Kynurenine/Tryptophan Ratio) and Kynurenine levels significantly in pancreatic cancer patients (Zimmer et al. 2019; Pal et al. 2021).

Data from various studies suggest that polarized endurance training (approximately 75% of sessions are performed at low, 10% at moderate and 15% at high intensities) might be superior to other endurance training methods, in terms of performance and overall health (Hydren and Cohen 2015; Laursen 2010; Stöggl and Sperlich 2014, 2015).

To our knowledge, no studies have investigated the effects of single bout of endurance exercise (acute) and chronic training of different endurance modalities (standard vs. polarized) on the AhR/IDO axis, especially in NK cells. Against this backdrop, we hypothesized that AhR and IDO levels as well as NK cell receptor expression are affected by acute exercise. Additionally, we hypothesized that polarized and standard training programs provoke different expression patterns of AhR, IDO, NKG2D and KIR2DL1.

## Methods

### Research design

This is a secondary analysis of the TOP study, a four-arm randomized controlled clinical training intervention trial aiming to identify the most effective training method in terms of enhancing physical fitness in breast and prostate cancer patients. The study is registered at ClinicalTrials.gov (NCT02883699) and was approved by the Ethics committee.

In this secondary analysis of the TOP study, we have focused on acute and chronic effects of endurance exercise. Before participants were randomized in one of two endurance training arms (polarized vs. standard), they performed a cardiopulmonary exercise test (CPET) to adjust training load. Before (pre-CPET), after (post-CPET) and one hour after (1 h post-CPET) the CPET blood samples were collected to determine effects of a single bout of exhaustive exercise (acute effects). Since all participants conducted the CPET, data are of longitudinal nature. After completing either the polarized or the standard twelve-week training, another resting blood sample (12 weeks) was taken to determine potential differences between both programs (chronic effects).

We report on acute exercise-mediated changes in NK cell receptors and AhR/IDO as well as chronic effects of 12 weeks of standard vs modified polarized endurance training on NK cell-mediated immune response in cancer patients. The serum samples were collected, frozen and stored at − 80 °C for our analysis. To determine changes in NK cells due to exercise, NK-92 (ATCC® CRL-2407™) cells were incubated with patient exercise serum. The incubated NK-92 cells were analysed via flow cytometry for surface markers as well as internal markers.

### Participants

In this randomized controlled training intervention study, 60 breast and 60 prostate cancer patients were recruited. Immune analyses were restricted to 21 subjects, including 9 participants from the endurance standard group and 12 participants from the endurance polarized group due to limited availability of the viable quantity of serum samples for all four-time points as well as serum that has not undergone haemolysis. Both informed and written consent was obtained from all participants. Criteria for inclusion were: patients diagnosed with non-metastatic breast cancer (M0) or non-metastatic or metastatic prostate cancer (M0 or M1, except for bone or brain metastases, with prostate-specific antigen evidence of stable disease), 6–52 weeks after the end of primary therapy (i.e., surgery and/or radiotherapy and/or chemotherapy), 18–75 years of age, and no regular vigorous training (> 1 session/week) since diagnosis or within the last 6 months. Exclusion criteria included: diagnosis with additional other cancer and severe comorbidities that precluded participation in exercise testing or training (acute infectious diseases, severe cardiac, respiratory, renal or neurological diseases).

### Intervention

#### Acute exercise (cardiopulmonary exercise tests)

CPETs were performed on a cycle ergometer. They began at 20 W and increased by 10 W every minute until patients reached volitional exhaustion. Then, following a 10-min resting period, each patient performed a supramaximal verification test at 110% of the previous CPET's peak power output (Schrenk 1998). Patients were advised to exert maximum effort once again, and the test was continued until volitional exhaustion was reached. More detailed information is given elsewhere (Schneider et al. 2020). Blood samples were taken immediately after cessation of the verification test. All patients were analysed together.

#### Chronic exercise (12-week training intervention)

Participants were randomly assigned to one of the following groups (stratification criteria: cancer type/sex, age and baseline fitness (VO_2peak_ in ml/min/kg body weight)):

(1) Standard endurance training group (*n* = 9): Two times per week 30 min of continuous cycle ergometer exercise at a vigorous intensity, slightly below (97%) the individual anaerobic blood lactate threshold (IAT). Intensity corresponding to this point was prescribed as power output (W).

(2) Polarized endurance training group (*n* = 12): Polarized training was modified for cancer survivors as follows: One time per week HIIT 4 times 4 min at 85–95% peak heart rate (HR_peak_) of the CPET and 3 times 3 min recovery at 70% HR_peak_ and one time per week moderate-intensity continuous training (MICT) at the first lactate threshold (LT1) (work rate-matched to the standard endurance training group, therefore duration was individual for each patient). All training sessions took place on a cycle ergometer.

### Blood sampling

Blood sampling was done by repeated venipunctures using a butterfly needle. Serum centrifugation was done by leaving the blood sample undisturbed for 30 min then centrifuging for 15 min at 2500 g (3700 rpm) at room temperature (acceleration and deceleration set at 9). S-Monovette® (Praxindo, GmbH; dimensions: 5 ml—Ø 15 × 92 mm) vacutainer was used for the collection of blood samples.

### Cell culture

NK-92 cells (ATCC® CRL-2407™) were grown through five passages, and then aliquoted and stored in liquid nitrogen to minimize target cell differences between assays. Frozen cells were thawed 2–10 days prior to each experiment and maintained in 2 mM L-glutamine and 1.5 g/L sodium bicarbonate enriched Alpha Minimum Essential medium without ribonucleosides and deoxyribonucleosides supplemented with 12.5% FBS (foetal bovine serum) and 12.5% horse serum as mentioned in ATCC guidelines. On every third day, the cells were fed with 50 IU of IL-2 (Prepotech catalogue number 200–02). On the day of NK cell assay, 1 × 10^5 target cells were removed and plated in 96 well (eBioscience, San Diego, CA, USA). Cells were incubated for 1-h with 50% autologous serum collected at the four different time points. This concentration of serum corresponds to the proportion of serum in whole blood in vivo and has been used in similar work before (Booth et al. 2010). Following incubation, cells were analysed on a BD FACSLyric Cytometer (Heidelberg, Germany).

### Flow cytometric analysis of NK cells

Serum incubated NK-92 cells were labelled with Fix/Via Stain 700(BD 564,997), PE-Cy7 conjugated anti-CD56- (eBioscience 25–0567-42), Alexa flour 647 conjugated mouse anti-IDO1 (BD 566,648), FITC conjugated KIR2DL1 (BD 556,062), BV421 conjugated mouse anti-AhR (BD 565,791), BV510 conjugated NKG2D (BD 563,266). All antibodies were purchased from BD Biosciences Heidelberg, Germany. Cells were incubated with 5 µL of each antibody for 20 min and 30 min for extracellular and intracellular staining, respectively, in the dark at room temperature, then washed and resuspended in 200 μl PBS prior to analyse on flow cytometry. Labelled cells were directly analysed with BD FACSLyrics cytometer. Live and dead cells were identified by Fix/Via stain through forward and side scatter characteristics and gated electronically using FlowJo™ software. CD56 + lymphocytes were gated as NK cells. The surface markers were identified by gating for NKG2D + , KIR2DL1 + and intracellular markers by AhR + , IDO + . We used one intracellular (IDO) and one extracellular (NKG2D) unstained marker for fluorescence minus two (FM2) control to determine the negative population. Representative plots demonstrating gating strategy are given in Appendix Fig. 1 (Supplementary file 1). The methodology and parameters used for flow cytometry data acquisition are listed in Appendix 1 (Supplementary file 1).

### Data analysis

All statistical data analyses were performed using IBM SPSS Statistics 25. At baseline, anthropometric and clinical outcome variables were compared between the groups, supported by using Pearson Chi-square test or unpaired *t* test. Data were analysed for normality by checking for skewness and kurtosis and were found to be normally distributed. To determine acute (CPET) effects over time, repeated measures ANOVA (pre-CPET, post-CPET and 1 h post-CPET) was conducted for expression of each outcome variable for all 21 patients. Significant time effects were further analysed by Bonferroni corrected simple effects analysis. To determine between group and within-group effects of different chronic endurance exercise over time, expressions were compared after randomisation using a baseline adjusted analysis of covariance (ANCOVA) for pre-CPET and 12 weeks after intervention for each group. Sex, age and treatments were not included as a covariate because initial analyses indicated they did not significantly contribute to variation. Prior to statistical testing, *Z* scores were calculated to identify possible outliers (> + /− 3 SD). We checked our data for sphericity using Mauchly’s test. In case of violation of sphericity, Greenhouse–Geisser correction was applied. For all analyses, the significance level was set at 0.05.

## Results

### Baseline comparison between different endurance exercise groups

Analysis of baseline differences for age (*p* = 0.72), weight (*p* = 0.151), BMI (*p* = 0.25) and cancer type (*p* = 0.55) between endurance standard and endurance polarized groups did not show any significance (mean data listed in Table 1).

**Table 1:** Anthropometric and clinical parameters of patient population

Study	Endurance polarized	Endurance standard
TOTAL (*n*)	12	9
Age (years), mean (SD)	60.67 (8.70)	59.11 (9.87)
BMI (kg/m^2^), mean (SD)	26.14 (3.53)	28.53 (4.01)
VO_2peak_ (ml/min/kg), mean (SD)	23.03 (3.68)	21.6 (4.71)
Breast cancer, *n* (%)	7 (58.33)	4 (44.44)
Prostate cancer, *n* (%)	5 (41.66)	5 (55.55)
Stage, *n* (%)		
I	9 (75)	7 (77.77)
II	3 (25)	2 (22.22)
III	0 (0)	0 (0)
IV	0 (0)	0 (0)
Treatment, *n* (%)		
Adjuvant hormone therapy	7 (58.33)	5 (55.55)
Antibody therapy	1 (8.3)	1 (11.11)
Neo-adjuvant chemotherapy	0 (0)	2 (22.22)
Intraoperative radiation therapy	1 (8.3)	0 (0)
Radiation therapy	9 (75)	6 (66.66)
Operative procedures, *n* (%)	10 (83.33)	8 (88.88)

*n* number of participants, *SD* standard deviation, *BMI* body mass index, *VO*_*2peak*_ peak oxygen uptake

### Acute effects of single bout of exercise on NK cells

**Fig. 2:**
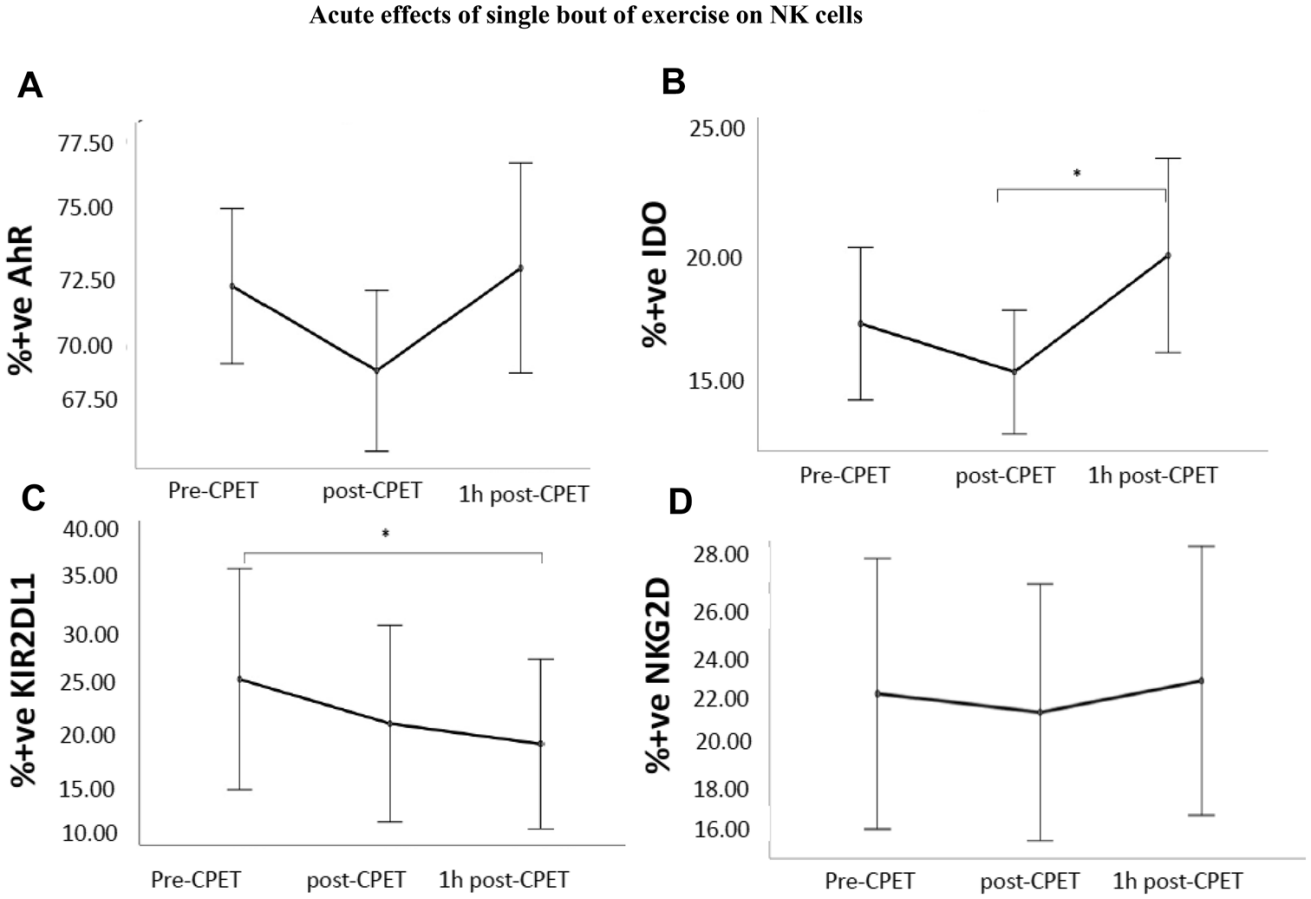
Acute effects of a single bout of endurance exercise: mean expression of AhR, IDO and NK cell receptors KIR2DL1 and NKG2D are plotted at pre-CPET, post-CPET and 1 h post- CPET. Data are presented as means with 95% confidence intervals. **A** AhR **B** IDO **C** KIR2DL1, **D** NKG2D (data listed in Supplementary Table 1). X-axis: time, Y-axis: % positive cells. *indicating results of statistical significance

Mean data and time courses of all outcome measures are shown in Fig. 2. For the receptor AhR, although expression was descriptively reduced post-CPET and was back to pre-CPET values after 1 h, ANOVA revealed no significant change over time (*p* = 0.13, *F* = 2.11, *df* = 1.78) (Fig. 2A).

For IDO, a significant change over time was observed (*p* = 0.02, *F* = 4.63, *df* = 1.52). Bonferroni corrected post-hoc analysis showed statistically significant increase between the following time points: post-CPET and 1 h post-CPET (*p* = 0.03) (Fig. 2B).

KIR2DL1 expression decreased significantly over time (*p* < 0.01, *F* = 6.15, *df* = 1.60) (Fig. 2C). Bonferroni corrected post-hoc analysis showed statistically significant decrease between pre-CPET and 1 h post-CPET (*p* = 0.03).

For NKG2D, the expression remained almost constant at all three-time points without any statistical significance (*p* = 0.31, *F* = 1.20, *df* = 1.69) (Fig. 2D).

### Chronic effects of different endurance training modalities on NK cells

**Fig. 3:**
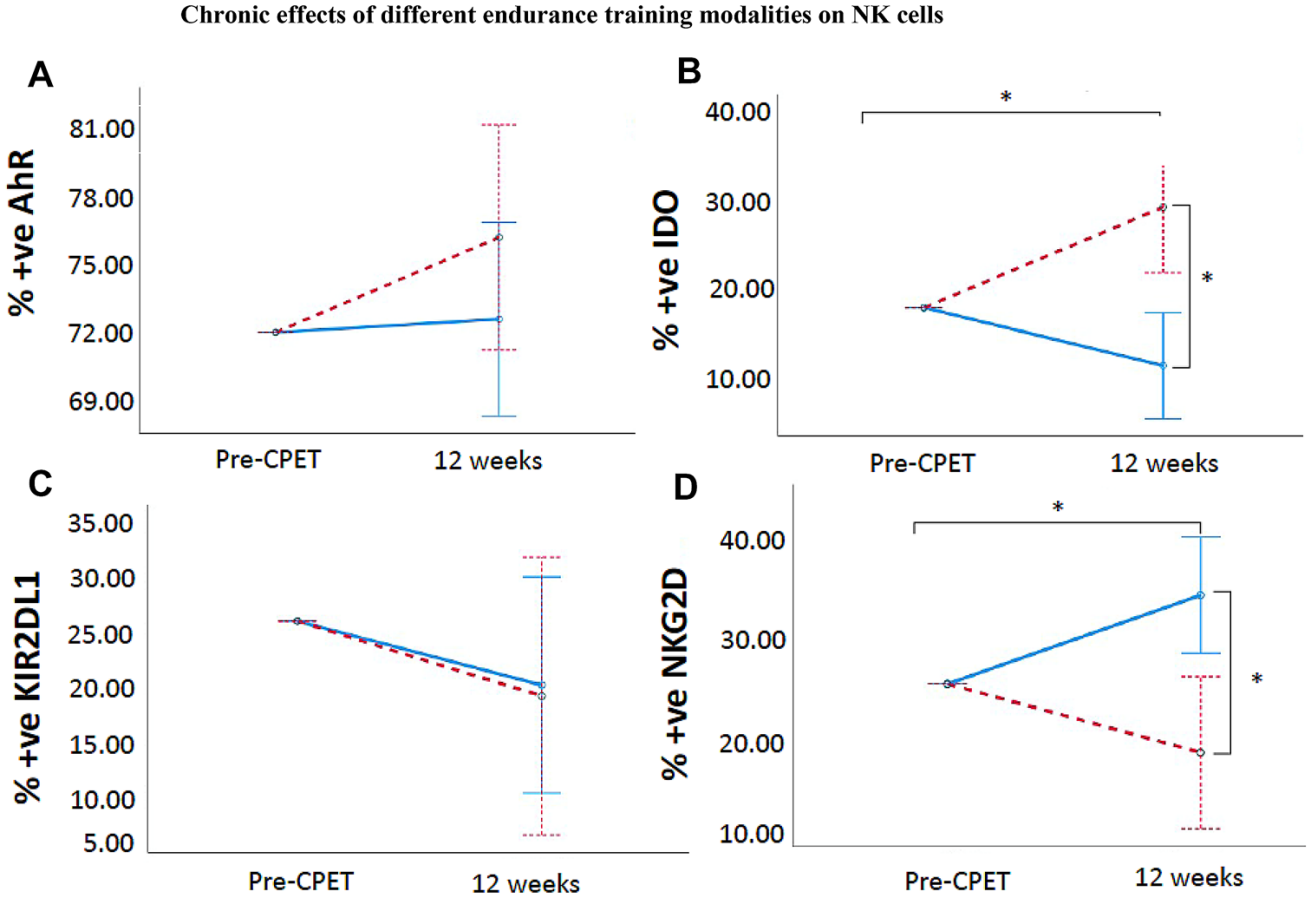
Chronic effects of different modalities of endurance exercise: baseline adjusted mean expression of AhR, IDO and NK cell receptors are plotted at pre-CPET and after 12 weeks of chronic exercise training modalities. Data are presented as means with 95% confidence intervals (endurance polarized: blue *n* = 12, endurance standard: dashed red *n* = 9). **A** AhR, **B** IDO, **C** KIR2DL1, **D** NKG2D (data listed in Supplementary Table 2). X-axis: time, Y-axis: % positive cells. *indicating results of statistical significance

For chronic effects of different endurance exercise modalities, the receptor AhR revealed no significant group × time interaction (*p* = 0.27, *F* = 3.59, *df* = 1.00) and no significant time effect (*p* = 0.07, *F* = 30.35, *df* = 1.00) was observed (Fig. 3A). For IDO, a significant group × time interaction was observed (*p* < 0.01, *F* = 10.66, *df* = 1.00) as well as a significant time effect was seen (*p* < 0.01, *F* = 10.03, *df* = 1.00). (Fig. 3B). Bonferroni corrected post-hoc analysis showed significantly increased IDO expression levels in endurance standard group compared to the endurance polarized group after 12 weeks of intervention (*p* < 0.01) but no significant within-group differences (*p* = 0.17). On the contrary, KIR2DL1 showed no significant group × time interaction (*p* = 0.92, F = 0.01, df = 1) as well as no significant time effect (*p* = *0.92, F* = *0.01, df* = *1*) (Fig. 3C). For NKG2D, we observed a statistically significant group × time interaction (*p* = 0.02, *F* = 6.45, *df* = 1.00), and a significant time effect (*p* = 0.03, *F* = 6.00, *df* = 1.00). However, Bonferroni corrected post-hoc analysis showed only significant result in between groups after 12 weeks (*p* = 0.02) and no significant within group differences (*p* = 0.27) (Fig. 3D).

## Discussion

To our knowledge, this is the first study that explored acute and chronic effects of endurance exercise in regards of AhR/IDO axis mediated changes in NK cell receptor expression in patients with breast and prostate cancer. Main findings of this study comprise that acute exercise leads to significant increases in IDO expression levels which are accompanied by significant decreases in the expression of the inhibiting NK cell receptor KIR2DL1. In regards of the comparison between the two different training programs, 12 weeks of polarized endurance exercise consequences in significant lower IDO expression levels compared to standard endurance exercise. Interestingly, the chronically reduced IDO expression in the polarized group was accompanied by a significant increase in the expression of the activating NK cell receptor NKG2D, whereas a contrary development was observed in the standard group.

Although not significant, the decreased levels of AhR immediately after CPET might be of importance as recent findings have revealed induction of IDO depends on AhR expression (Nguyen et al. 2014). Consequently, IDO-mediated Tryptophan catabolism and Kynurenine formation which is an AhR agonist is an important immunoregulatory mechanism underlying immunosuppression and tolerance (Park et al. 2019). Despite NK cells’ ability to directly eliminate cancer cells, they are inhibited by several factors, one such factor being IDO mediated suppression (Wang et al. 2012; Hornyak et al. 2018). Therefore, acute single bout of endurance exercise may reduce AhR levels as well as IDO levels in NK cells. It can be worth speculating that whether the detected decrease in AhR levels in the cytoplasm post-CPET is due to lower expression or whether AhR translocate more into the nucleus forming the transcriptional activation complex. The similar expression pattern of both AhR and IDO is indicative of the inter-dependent relationship between them. The restored levels of AhR and IDO after 1 h of CPET can indicate to the AhR and IDO feedback loop (as discussed in Introduction section (Nguyen et al. 2014)), as IDO levels correspond to the AhR expression pattern forming the AhR/IDO axis.

To understand whether the change in expression of AhR and IDO directly affects NK cell activating and inhibiting receptors, we checked for surface makers of NKG2D and KIR2DL1. The significantly overall reduced expression of inhibiting receptor KIR2DL1 as well as the significant reduction between pre-CPET levels and 1 h post-CPET levels would mean an absence of NK cell-specific inhibitory signalling cascade. Although the activating receptor NKG2D expression is affected in a dose–response manner (shown previously by Zimmer et al. 2015), it is interesting to note that the expression does not change. It can be speculated that probably the single bout of endurance training performed was not of enough stimuli to change the receptor expression. It must be emphasized that we assessed NK cell receptors, but to establish a clinical relevance on NK cell cytotoxicity, NK cell cytotoxic assay should have been performed.

After randomisation into two different endurance training modalities our results on chronic effects of endurance training over 12 weeks were equally pronounced. The change in expression of AhR levels was not affected significantly after 12 weeks of polarized or standard endurance training. It is of importance to note that although the difference is not of significance, the change over 12 weeks of AhR levels were slightly higher in the endurance standard group compared to endurance polarized group. It is interesting to note IDO levels after 12 weeks of training differed significantly between both groups, with a higher expression in endurance standard group. The expression of KIR2DL1 was non-significantly decreased within both endurance polarized and standard groups after 12 weeks of intervention, thereby possibly interjecting the inhibition cascade. The most promising results are of NKG2D expression as it was significantly higher in the endurance polarized group compared to the endurance standard group after 12 weeks of intervention. This again points to the fact that endurance polarized exercise which contains high-intensity interval training has different effects compared to standard training.

Therefore, we can conclude that irrespective of acute or chronic phases, KIR2DL1 expression might be lowered by endurance training. Acute exercise has more pronounced effect on all analysed outcomes in contrast to chronic exercise. Chronic polarized exercise has a potential to downregulate the AhR/IDO axis and activate NK cells cytotoxicity. We can also concur that endurance polarized training might represent a more potent stimulus for AhR/IDO-mediated NK cell receptor changes compared to a standard endurance exercise regime. Our results suggest under acute endurance exercise, IDO levels are lowered immediately post-CPET but increase 1 h post-CPET significantly. Previous studies have reported that IDO levels increase with acute exercise and decrease after chronic exercise (Metcalfe et al. 2018). This can be since most studies measure IDO as an indirect measure of Kynurenine and Tryptophan ratio. It is a possibility that free Tryptophan is being utilized for protein synthesis after a vigorous exercise session. Repeated bouts of intense exercise exert a higher demand for protein synthesis thereby, decreasing the amount of available Tryptophan to be degraded into Kynurenine, consequently leading to a lower Kynurenine/Tryptophan ratio. As no studies till date have investigated the effects of endurance exercise in its both acute and chronic phases, it is possible that under higher energy demand, the Tryptophan breakdown is halted.

Our findings are a first step in understanding how the AhR/IDO axis may modulate NK cell function via exercise. It has already been established that within tumor cells, IDO converts Tryptophan to Kynurenine (see Introduction). Increased levels of Kynurenine in the microenvironment can enter NK cells and subsequently, act as a potent internal AhR agonist. Upon ligand binding, the AhR-Kynurenine complex translocate into the nucleus where it binds to the DREs (Dioxin responsive elements). This acts as a transcriptional regulator and leads to the production of further IDO and increases surface KIR2DL1 (inhibitory) and decreases NKG2D (activating) expression. According to our results, endurance exercise might lower IDO levels and reduce AhR agonist Kynurenine production. This suppresses the downstream cascade of AhR ligand binding and its transcriptional activity. Hence, expressing lower KIR2DL1 and higher NKG2D levels, leading to enhanced NK cell cytotoxic potential.

The findings of the present study should be read within the context of its strengths and limitations. Although we are limited by a small sample size (*n* = 21), this is a unique study exploring different endurance training modalities on NK cell activation via AhR/IDO axis in cancer patients. Another limitation includes clustering in KIR2DL1 and NKG2D markers, therefore the analysis should be considered exploratory. Further, to understand the expression of these markers, protein quantification techniques could be useful. To understand how the AhR/IDO axis functions specifically in NK cells, further investigation into Kynurenine transporters as well as other Kynurenine pathway metabolites are imperative. It would also be interesting to check for AhR levels in the nucleus to understand whether exercise leads to the translocation of AhR from the cytoplasm. Future investigations should also focus on determining whether different resistance exercise modalities have similar potential to modulate the AhR/IDO axis leading NK cell function.

We conclude that both acute and chronic endurance training has the potential to regulate NK cell function via the AhR/IDO axis. This is of clinical relevance as promising cancer therapeutics based on the intervention of the AhR/IDO axis are already under extensive investigation (Nguyen et al. 2014; Bianchi-Smiraglia et al. 2018). Harnessing the body’s potential for exercise can mimic immune therapy and may lead to better health outcomes.

Although all the experiments were performed and analysed simultaneously, there was some clustering observed in KIR2DL1 and NKG2D marker. This is hard for us to explain as no other marker showed any clustering or batch effect. Therefore, the analysis should be considered exploratory and this should be taken into consideration while interpreting the results.

## Supplementary Information

Below is the link to the electronic supplementary material.

Supplementary file1 (DOCX 1216 KB)

## Abbreviations

NK Cell: Natural killer cell
AhR: Aryl hydrocarbon receptor
ANCOVA: Analysis of covariance
ANOVA: Analysis of variance
BMI: Basal metabolic index
CPET: Cardiopulmonary exercise test
DRE: Dioxin-responsive elements
FBS: Foetal bovine serum
FM2: Fluorescence minus 2
HIIT: High-intensity interval training
HLA: Human leukocyte antigen
HRpeak: Peak heart rate
IAT: Individual anaerobic threshold
IDO: Indolamine 2,3–dioxegenase
KIR: Killer cell immunoglobulin-like receptors
KIR2DL1: Killer cell immunoglobulin like receptor, two Ig domains and long cytoplasmic tail 1
LT1: First lactate threshold
MHC: Major histocompatibility complex
MICT: Moderate intensity continuous training
NKG2D: Natural killer group 2 member D
SD: Standard deviation
VO2peak: Peak volume oxygen
