# Supplementary material for: Different endurance exercises modulate NK cell cytotoxic and inhibiting receptors
Source: Eur J Appl Physiol. 2021 Sep 3;121(12):3379–87. doi: 10.1007/s00421-021-04735-z (PMC8571223; doi:10.1007/s00421-021-04735-z)
Supplement: Supplementary file 1 — Supplementary file1 (DOCX 1216 KB) [file 421_2021_4735_MOESM1_ESM.docx]

**Supplementary data and figures**

**Supplementary Table 1: Acute endurance effects on NK cell outcomes at pre-CPET, post-CPET, 1h post-CPET. Absolute mean values and standard deviations (SD) are listed for all outcomes (significant values are in bold).**

**Supplementary Table 2: Chronic endurance effects on NK cell outcomes between pre-CPET and 12 weeks. Mean values and standard deviations (SD) are listed for all outcomes (significant values are in bold).**


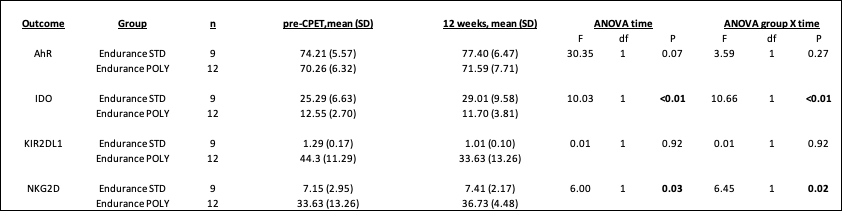


**Appendix 1**

**Figure 1**

Representative plot of gating strategy for a single patient. A-Histogram, B- dot plots. NK-92 cells were analysed by flow cytometry, gating by forward/side scatter. The markers used: NKG2D, KIR2DL1, AhR and IDO at four different time points (T0-pre-CPET, T1- post-CPET, T2- 1h post-CPET, T3-12 weeks).


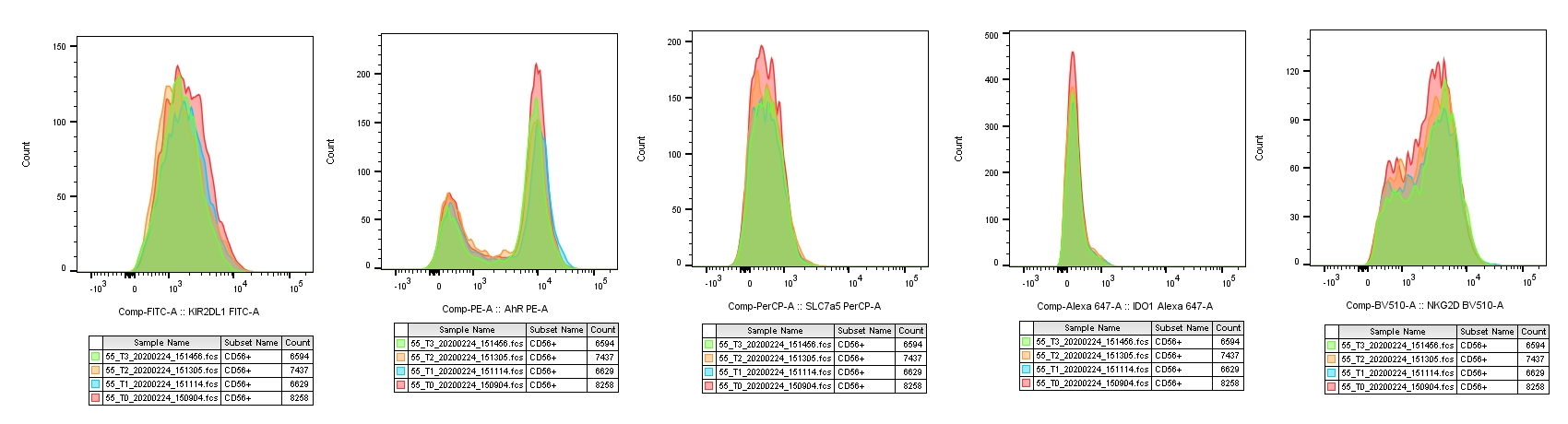


A


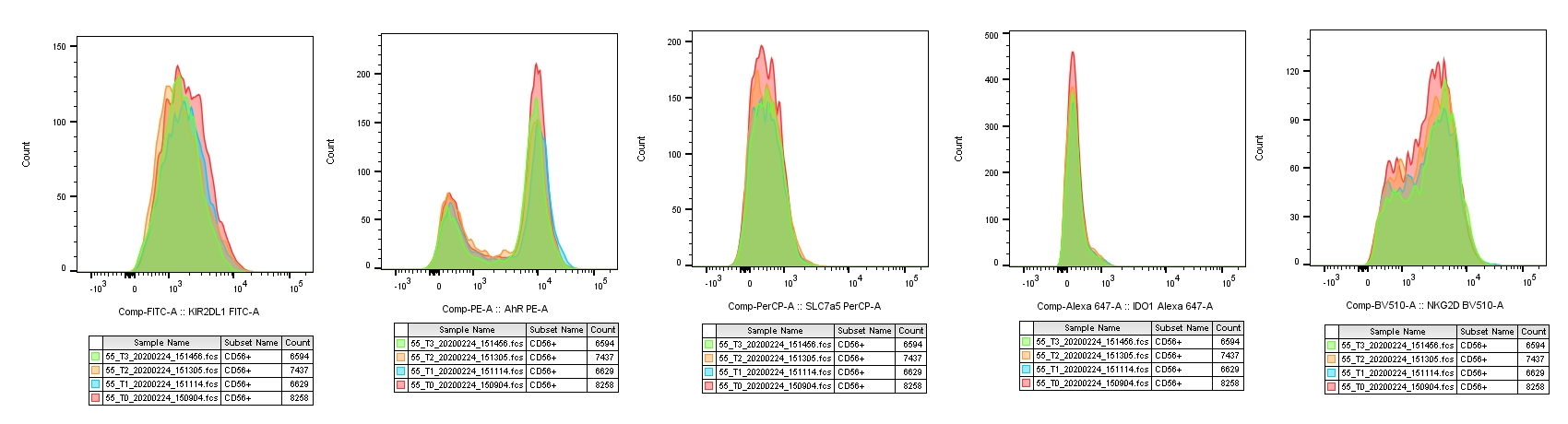


B


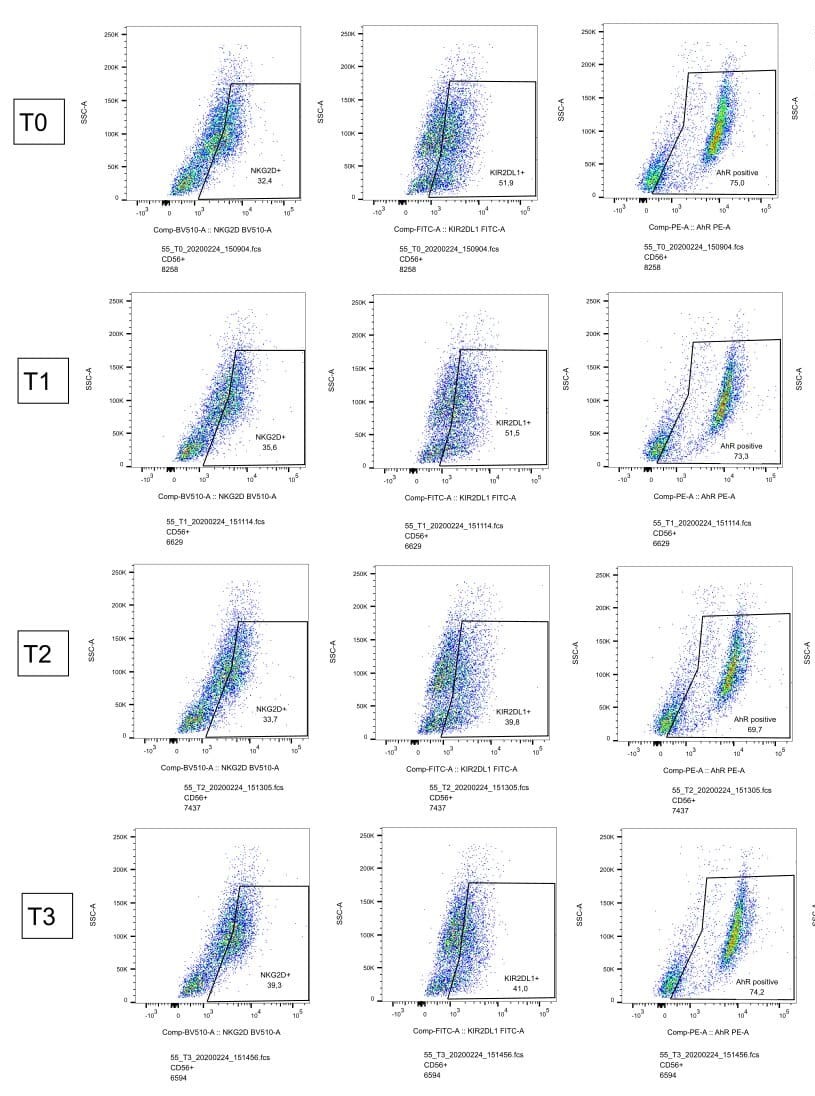


T0

T1


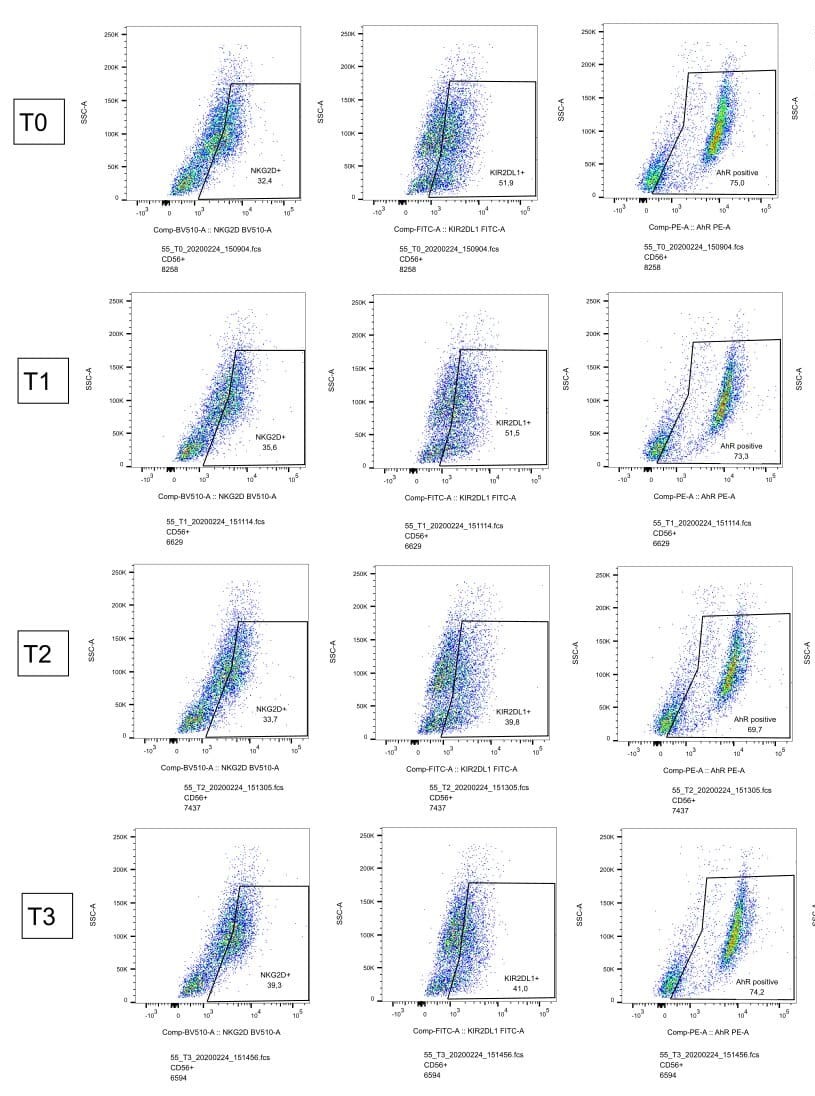


T3

T2


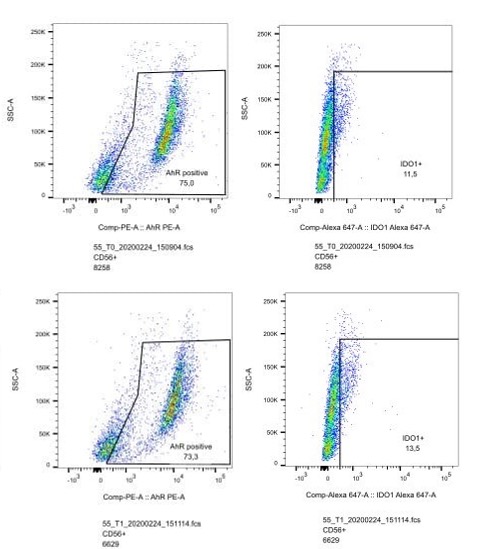


T1

T0


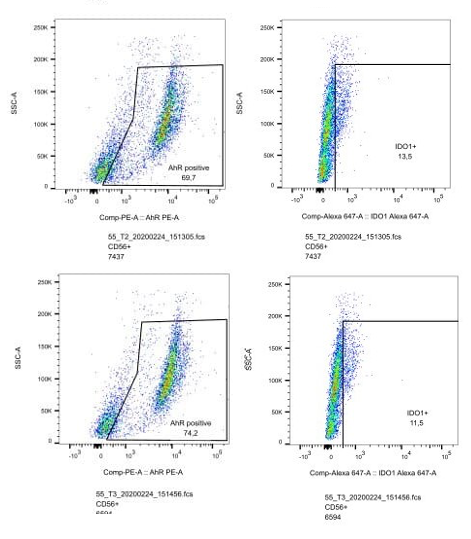


T3

T2

The methodology and parameters used for flow cytometry data acquisition

**
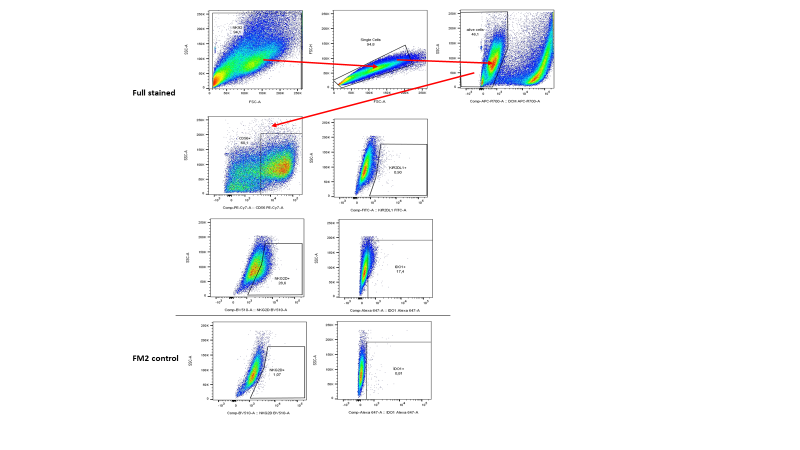
**

**Flow cytometer settings**

The BD FACSLyric™ system comprises a flow cytometer available in different optical configurations with BD FACSuite™ Clinical software, and optional BD FACS™ Universal Loader. By using patented BD™ FC Beads and BD CS&T Beads a universal setup for performance QC, instrument control, data acquisition/storage, online/offline data analysis, and instrument standardization can be ensured.

Our system consists of 14 parameters in total, 12 colours across up to 3 Lasers plus the parameter for cell size (FSC) and granularity (SSC). The laser configuration is 4 blue, 3 red and 4 violet lasers. The PMT voltages are automatically updated to maintain target MFI values as a part of Quality control every 60 days only. This daily bead-based Setup ensures <0,4% variability across all parameters which leads to reproducible results. The 20 min compensation procedure only needs to be performed every 2 month and the spill over values (SOVs) are automatically updated as part of daily quality control.

**Flow cytometry data acquisition**

The raw data can either be analysed directly on the FACS Lyric by using the BDSuite Software, which is also the measure software of the samples or can be exported as fcs files into the Flow Jo Software (BD).
